# Performance Alteration Induced by Weight Cutting in Mixed Martial Arts—A Biomechanical Pilot Investigation

**DOI:** 10.3390/ijerph19042015

**Published:** 2022-02-11

**Authors:** Yufeng Liu, Jared Evans, Jacek Wąsik, Xiang Zhang, Gongbing Shan

**Affiliations:** 1 Department of Physical Education, Xinzhou Teachers’ University, Xinzhou 034000, China; liuyf@xztu.edu.cn (Y.L.); zhangxiang@xztu.edu.cn (X.Z.); 2 Biomechanics Lab, Faculty of Arts & Science, University of Lethbridge, Lethbridge, AB T1K 3M4, Canada; jared.evans@uleth.ca; 3 Department Kinesiology and Health Prevention, Jan Długosz University in Czestochowa, 42-200 Czestochowa, Poland; jwasik@konto.pl

**Keywords:** striking power, striking accuracy, reaction time, 3D motion capture, EMG measurement, biomechanical modeling

## Abstract

Currently, there are pros and cons of research results related to weight cutting in combat sports, resulting in inconclusive results regarding the effects of weight-cut on athletes’ performance, and biomechanical investigations are hardly seen. Therefore, this pilot study tried to fill the gap by initiating an exploration in real-life competitions. It is our hope to add biomechanical insights (advantages/disadvantages) that would discern the impact of weight cutting on competitive performance and help to structure hypotheses in future research. The method consisted of 3D motion capture, EMG measurement and biomechanical modeling. Through the synchronized data, striking power, striking accuracy and reaction time were quantitatively determined. Pre- and post-test design was used to test common strikes before weight cutting and 24 h after weigh-in. Seven male athletes from local clubs were tested during regional competitions. Results were characterized by using descriptive statistics (means and standard deviations) and T-tests were performed to contrast differences between the pre- and post-tests. This pilot study has revealed that there is actually weight-regain instead of weight-loss. The weight-regain would speed up the perceptional and total reaction, slow down the limbs’ movement, worsen the striking accuracy and, possibly, decrease the strike power. The preliminary results are inconclusive regarding the competitive advantages/disadvantages induced by weight cutting. Further biomechanical studies are needed to deal with the controversial subject more objectively and scientifically.

## 1. Introduction

In combat sports, competitors are divided into various weight classes to ensure fair competitions. As a direct consequence, weight cutting is common in these sports in which athletes seek exclusively to compete one or more weight classes below their normal weight [1,2]. The common way for reaching the goal is to rapidly lower the weight through a combination of strategies that often involve dehydration prior to weigh-in. After the weigh-in with dehydrated bodies, the athletes will then eat and rehydrate for quick increases of their weight.

The common belief behind weight cutting is that it would give the weight-cut athletes advantages in the competition. Yet, the so-called advantage of weight cutting is offset by the fact that the combatant’s opponent has most likely done the same. Of course, then the argument for using weight cutting is that the athlete must use them because his opponent is using it. It seems to be logical that weight cutting becomes a part of a subculture of these sports and, as such, is considered “normal.” Studies have shown a high to very high prevalence of weight cutting (60–90% of competitors) among high school, collegiate and elite combat athletes [3,4,5,6].

Weight cutting is not without health risks. Empirical evidences document injuries related to the side effects of weight cutting, such as dizziness, lightheadedness, fast fatigue, and even deaths resulting from rapid extreme weight cutting [7,8]. The empirical evidences clearly indicate that weight cutting is putting athletes at significant danger. Yet, mixed results of scientific studies in the past three decades have been inconclusive. There are investigations unveiling that weight cutting practices can negatively influence athletes’ performances [9,10,11,12,13], while others reveal no impact [14,15,16,17,18] or even improved performances [19,20,21,22]. The negative influences include decreased short-term memory, vigor, concentration, and self-esteem, as well as increased confusion, rage, fatigue, depression and isolation [9,11,12,13,23]. On the other hand, contradictory results claim that rapid body mass loss does not impair performance in combat athletes; some studies have even found that body mass recovered after the weigh-in leads to successes in competitions [19,20,21,22]. The current scenario indicates that weight cutting is a very complicated issue influenced by multi-layer factors and its impacts on physical performance are scientifically not sufficiently demonstrated. As a consequence, many athletes accept the culture of privileging winning the game over their long-term wellness [7]. Obviously, weight cutting is a practice that is not going away any time soon. Many more scientific studies are needed to quantify the phenomenon holistically, i.e., the competition advantages related to physiology, biochemistry, psychology, biomechanics, and more.

It is worth noting that, in the past three decades, studies have overwhelmingly investigated physiological, biochemical and/or psychological parameters. Quantifications on biomechanical influences are difficult to find. A search using the key words “combat sport + weight cut + biomechanics” in Web of Science in December 2021 returned zero records. Without biomechanical quantification, an understanding of the effects of weight cutting remains incomplete for combat sports.

Previous studies have shown that biomechanical parameters, such as attack power [24,25,26,27,28,29,30,31], attack accuracy [24,26,31] and reaction time (time for preparing and performing an attack) [24,25,29], are directly linked to competitive successes. Biomechanically, attack power (i.e., explosive strength) is used to characterize an athlete’s ability to produce as much force and velocity in as short amount of time as possible. It is a vital element for any athlete seeking to become better in combat sports. Attack accuracy (i.e., precision of segments/joints’ coordination) represents another vital ability of martial-arts athletes. A decreased attack accuracy during fighting results in missing the target more often than not, and thus the athlete fails to accrue either decisive strikes on the opponent or points from the judges. Reaction time demonstrates how fast an athlete can response to a dynamic situation that he “reads” in fighting. Agility, quickness and speed are mainly what will determine the quality of a reaction. Together, attack power, attack accuracy and reaction time refer to the actual fighting ability and, therefore, the crucial determinants of competition successes.

Consequently, in order to quantify the possible advantages/disadvantages induced by weight cutting, it is important to make use of these biomechanical parameters for gaining vital evidences. The current literature search shows that there is dearth of studies investigating these parameters related to weight cutting in combat sports. Additionally, recent review articles have unveiled that, among the numerous studies in the past, there are limited investigations quantifying the effects of weight cutting in real tournaments [1,3]. Hence, the current study tried to fill these gaps by initiating a biomechanical analysis in a real-life competition environment. It is our hope to add biomechanical insights (advantages/disadvantages) that would discern the impact of weight cutting on competitive performance and help to structure hypotheses in future research.

## 2. Materials and Methods

A central goal of this study has been to investigate the weight cutting phenomenon in its real life setting. Therefore, the competition setting was used to avoid laboratory-based investigation, i.e., to gather the selected biomechanical data in close to its real sport setting. A method developed for determining power and neural responses in a martial arts quasi-training environment [24] was applied. The data collection using this method is very close to the real-life condition with neglectable test influence on an athlete’s performance. 

### 2.1. Subjects

The current study recruited amateur male MMA athletes from local clubs scheduling for actual regional competitive events. Because the test/data collection was relatively time-consuming (about two hours, one hour for the pre- and the other for the post-test), this factor clearly influenced the participation willingness of many athletes. In order to initiate the pilot study, the only inclusion criterion applied in the current study was the participation willingness. For promoting the participation willingness, our team used all contact possibilities to make personal communication with athletes registered in the regional competitions for showing them the practical significance of this scientific research, i.e., introducing biomechanical factors that they should know and consider for preparing their competitions. With our great effort, 12 athletes were successfully recruited, signed the consent forms and voluntarily participated in the study. Among the 12 subjects, five were unfortunately excluded. The only exclusion criterion was if the athlete was unable to complete the post-test. The main reason for this was conflicts between the test and personal preparation for the competition. Table 1 shows the subjects’ information that is relevant to this study. The host university scrutinized and approved the test protocol as meeting the criteria of ethical conduct for research involving human subjects.

### 2.2. Synchronized 3D Motion Capture and Full-Body Biomechanical Modeling

The method consists of 3D motion capture and EMG measurements as well as biomechanical modeling of a standard punching bag and an athlete’s body. Three systems—a visual stimulus system with three LEDs, an eight-channel wireless EMG (NORAXON USA. Inc., Arizona, USA) and a twelve-camera VICON 3D motion capture system (Oxford Metrics Ltd., Oxford, England)—were synchronized for biomechanical data collection.

The LED and EMG systems were used to determine the neural response of an athlete’s strikes. The EMG data were wirelessly recorded at a rate of 1000 Hz with a band pass filter of 16–500 Hz. The EMG electrodes were positioned over the ‘belly’ of eight selected muscles: left and right biceps, triceps, quadriceps and hamstrings. This setting allows an investigation of muscular recruitment, activation and onset differences [24]. The use of a wireless EMG system minimizes the constraints that normally occur with the use of wired electrodes, where subjects’ dynamic movements and motor control patterns may be inadvertently altered due to the presence of wires connecting the subject to the measuring system. The LED system supplies a stimuli for initiating a striking action. Together with the EMG measurement, the amount of time that has elapsed between the stimuli and the response to that stimuli (i.e., the reaction time) is quantitatively determined [24].

**Figure 1:**
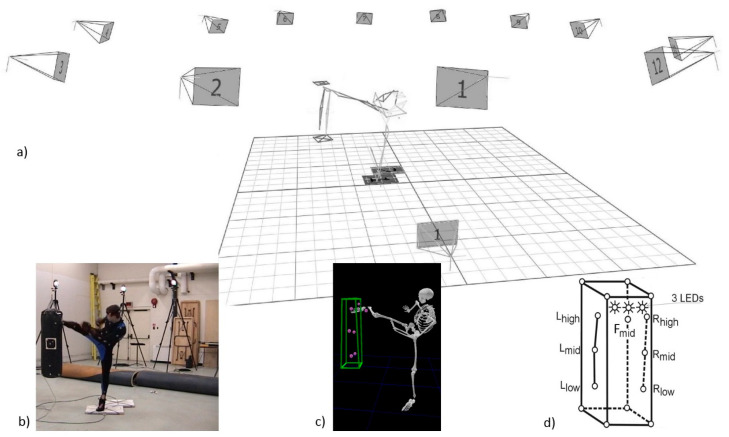
The synchronized data collection: (**a**) the set-up of the data collection; (**b**) a sample frame; (**c**) the biomechanical modeling based on 3D motion capture data; (**d**) the set-up of the target markers and LEDs on the bag.

The stimulus system comprises three LED lights and is placed at the top of the bag. One of the LEDs (a stimulus) is initiated by a remote trigger controlled by the researcher. The stimulus system is used to initiate the time of strikes/punches, as well as to indicate to the subject the location of strikes/punches [24]. For example, the researcher informs the subject that the next trial is a right kick, which enables the subject to be ready and then randomly initiates a LED (i.e., a stimulus): left LED—kick on head, middle LED—kick on body and right LED—kick on leg. The initiated LED is used to indicate to the subject the start of the trial and synchronized data collection is started at the same time. In this study, the system provided the following signals: straight, left or right for punches, as well as head, body or leg for kicks. These strikes were randomized by the researcher (Figure 1d).

The 3D motion capture and biomechanical modeling were used to quantify striking power and accuracy [24]. The use of motion-capture permits allowed considerable freedom of movement without influencing the accuracy of data [32,33,34,35], so no restrictions were placed on the movement of the subject within the capture volume and subjects could perform without having to alter their normal body control. Using the manufacturer’s specified guidelines, calibration resolution yielded results that were accurate within 1 mm. Data were gathered at 200 frame/s, which allowed the finest details of movement to be accurately examined.

During the 3D motion data collection, 39 reflective markers with a diameter of 9 mm were placed on each subject at specific bony landmarks (Figure 1a,b). Using a biomechanical model, as in previous studies [25,32,33,36], a 15-segments full-body model was created for striking quantification (Figure 1c). The anthropometric data required for the full-body model were estimated using anthropometric norms found in previous studies [37,38]. Additionally, a standard punching bag was outfitted with fifteen markers; eight of these were located on the top and bottom of the bag to frame the bag, and the other seven markers were located as follows: three at the vertical left, three at the vertical right and one at the front (Figure 1c,d). The eight markers that modeled the bag were used to determine striking power [24]. The other seven markers corresponded to the striking targets which were used to measure the striking accuracy. 

### 2.3. Quantification of Biomechanical Parameters

Neural response/reaction time, striking power and striking accuracy are the fundamental elements in many martial arts competitions [24,25,26,27,28,29,39]. These biomechanical parameters were quantitatively determined using synchronized data and the method proposed by Chang et al. [24].

**Figure 2:**
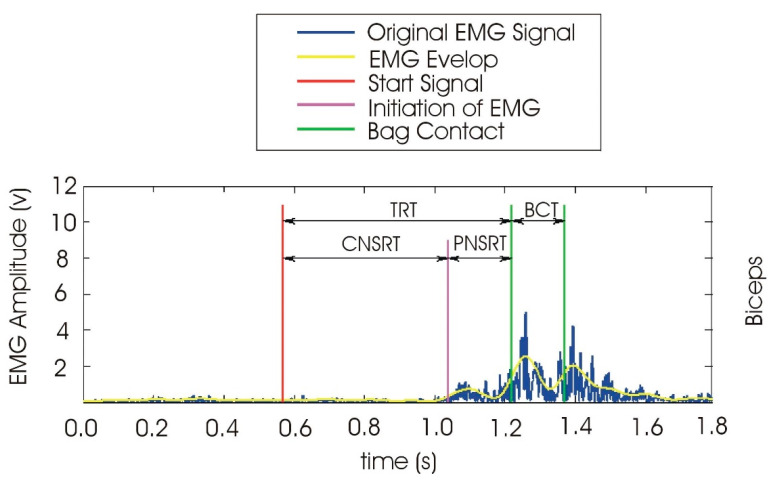
Quantification mechanism of the reaction time using the synchronized data collection and EMG enveloping method [24]. TRT: total reaction time, CNSRT: central nervous system response time, PNSRT—peripheral nervous system response time, BCT: bag contact time.

The reaction time consists of two parts: the central nervous system response time (CNSRT) and the peripheral nervous system response time (PNSRT). CNSRT refers to the time from a stimulus to the beginning of muscle movement, while PNSRT is related to the movement time of limbs, i.e., the time from the beginning of muscle movement to the completion of contraction [24,40]. Figure 2 shows the determination of the reaction time and its components using Chang et al.’s method. 

Every strike produces a unique movement of the punching bag with two components: translation and rotation. Treating the punching bag as a rigid body and utilizing the 3D data of the eight bag frame markers, linear power and angular power were able to be calculated and, hence, the total striking power could be calculated as well [24]. The linear power is related to the translational movement of the bag after striking, while the angular power is the cause of the rotary movement of the bag. Additionally, the peak power (max) and average power were commonly used to characterize the explosive degree of a strike. Hence, Chang et al.’s method was applied in quantifying these characteristics of the striking power in this study.

The seven target markers on the bag, combined with the carefully placed striking markers on the middle knuckle of the glove and lower shin, allowed for an investigation of striking accuracy. The accuracy was determined by quantifying the minimum distance between the appropriate target and strike marker using the distance equation:

d = √ [(x_t_ − x_s_)^2^ + (y_t_ − y_s_)^2^ + (z_t_ − z_s_)^2^] (1)

where (x_t_, y_t_, z_t_) and (x_s_, y_s_, z_s_) are the 3D position data (i.e., coordinates) of the target and the strike marker respectively.

### 2.4. Protocol

Pre- and post-test design was applied to investigate the effect of a weight cutting regimen on the biomechanical parameters in mixed martial arts (MMA) competitions. A variety of common strikes were selected for the test: strikes with the fist—right and left straights to the head, right and left hooks to the head, and right and left hooks to the body; as well as strikes with the foot—right and left round-house kicks to the head, body, and legs. For each target, the subject performed five strikes. The tests were randomized in the following categories: straights to the head (two LEDs used: left or right), hooks to the head (two LEDs used), hooks to the body (two LEDs used), left kicks (three LEDs used: head, body or leg), and finally right kicks (three LEDs used). The test resulted in 60 trials per subject.

In order to take the advantage of the method (i.e., the quasi-real-life condition with neglectable test influence on the athlete’s performance), no restrictions were placed on subjects’ weight-loss protocols; the athletes were allowed to utilize their preferred methods and timing strategies of weight reduction and regain. This approach preserved the tested subjects’ normal “style” in competitive preparation and made the results more realistic. The pre-tests were done in the evenings, about the same time of day of the planned competitions. Before the biomechanical tests, the initial weights of the participants were recorded. After the pre-tests, the participants selected their own ways to complete weight cutting for achieving their desired weight (i.e., the necessary weight class). Individually, the process varied from one to seven days. The subjects were post-tested after a 24 h period (MMA rule) of rehydration and re-feeding as close to their real competitions as feasible. Weigh-in and post-test weights were also recorded for our analysis. 

### 2.5. Data Analysis

All parameters obtained from the pre- and post-tests were analyzed using SPSS (IBM SPSS Statistics, version 26, Coppell, TX, USA). Due to indeterminacies resulting from the very small sample size (seven subjects) with a large between-subject deviation due to the confounding variables—such as weight class, amount of weight cut, duration of weight cutting, and dominant/non-dominant strikes—the current study is essentially a case study with a number of observations, rather than a statistical study. One common practice in case studies is to use individual data for revealing potential trends/influences and to apply descriptive statistics, i.e., mean (µ) and standard deviations (SD), to illustrate the characteristics of the selected parameters. In order to identify the potential influences (advantages/disadvantages) that exist in the individual data, the statistically defined majority (i.e., 68%) was applied as a cut-off criterion, i.e., a parameter is identified as a potential one when five or more out of the seven subjects show its increase/decrease. The advantage (position effect) represents a selected biomechanical parameter that is improved by the weight cutting process; otherwise it is the disadvantage (negative effect). Because of the selected sample size (i.e., five to seven) for the identification of potential performance changes induced by weight cutting, the Shapiro–Wilk Test was first performed to test the normality of the potential parameters, if normal, the paired T-test between the pre- and post-test was conducted to detect changes and the significance level was set at *p* = 0.05. Additionally, where there was a significant change, the percentage difference between the pre- and post-test was calculated using the formula (post-value − pre-value)/pre-value for indicating the degree of weight cutting influence on MMA competition, i.e., advantage/disadvantage.

## 3. Results

The weight changes during the individualized weight cutting process are presented in Table 2. On average, the amount of weight lost was 3.5 kg or 4.5%. Weight regain during the 24 h interval after the weigh in was 4.2 kg or 5.4%. All but one participant competed at a higher or about the same body weight when compared to their pre-cutting weight. 

Table 3 shows the influence of individualized weight cutting processes on individual performances. The results indicate that consistent changes (≥five subjects) are found in the parameters related to the reaction and accuracy of MMA strikes, but not in the parameters related to the power of the strikes. Specifically, the weight cutting process shows a positive effect on CNSRT and the total reaction (faster), and a negative effect on PNSRT (slower) and on accuracy (less accurate) of the majority of the subjects. Further, the weight cutting process is linked to more negative effects on power parameters (57% of 42 selected power parameters). In addition, the actual weight-regain subject (i.e., a higher body weight when compared to one’s pre-cut weight) displays more positive effects (e.g., subject 1 and subject 6) than the actual weight-loss subject (e.g., subject 7). 

The overall effect of weight cutting, i.e., a comparison of all of the trials for all strikes by all subjects, pre- versus post-test, is characterized by using descriptive statistics (µ ± SD), as seen in Table 4. The Shapiro–Wilk tests were performed on the potentially affected parameters and confirmed the normality of the reaction and accuracy data (*p* > 0.10). The T-test revealed that significant differences existed in terms of the reaction time and striking accuracy. The weight cutting made the CNSRT and total reaction faster, while the movement of limbs became slower (PNSRT), and strikes became imprecise. The CNSRT and total reaction time were 18.52% and 6.25% quicker than normal, respectively. The limbs’ movement was 19.23% slower than normal and the striking accuracy decreased by 7.46%. 

## 4. Discussion

To the best of our knowledge, this study is the first application of biomechanics-related weight cutting research to ongoing real-life phenomenon. The aim of this study has been to supply biomechanical evidences for quantitatively identifying the advantages/disadvantages yielded by weight cutting, and establish possible causal-effect connections between performance quality and weight cutting where possible. It seems that this preliminary study suggests more questions than answers. The following are some aspects worthy of discussion.

It is surprising to see that, contrary to common expectation, six out of seven subjects could return to their pre-weights or even regain more weight than their normal weight prior to their competitions. Our result is not an isolated one. A recent study in 2019 has reported the same phenomenon [21]. It is should be noted that the subjects of both studies are MMA athletes. It seems that weight cutting would create a strength advantage for MMA athletes based on the weight regain (i.e., a single-factor analysis). However, this judgement could not be so simply made biomechanically, because weight regain does not necessarily equate to the power advantage. The preliminary results would reject such a single-factor judgement. Overall, there was no significant difference in the strikes’ power generation between the pre- and the post-test. Yet, the power generation of three striking styles, i.e., the left straight to the head, the left and the right kick to the body, were significantly decreased (Table 4). The decreases ranged from 10% to 63%, indicating the possibility that weight return to normal/higher would not fully restore, and may even worsen, the strength ability. Of course, more studies are needed to verify the probability of what occurred in this preliminary study.

Further, it is of interest that there was a positive effect on the total and central reaction but a negative effect on the peripheral reaction. The result would suggest that weight cutting (especially, the positive weight regain) would speed up the judging/perception process, while slowing down the limbs’ movement. Since the judgement (CNSRT) takes about double the amount of time as the movement (PNSRT), the total effect on the reaction is positive, i.e., the strikes of athletes with more weight regain have been sped up. This result might indicate an unveiled/unknown advantage induced by weight cutting. Future studies are absolutely needed to check if the positive effect can be generalized or can only be repeated in the positive weight regain.

In practice, one of the most common ways to evaluate a fighter is to consider his/her striking accuracy [41]. The effect of weight cutting on strike accuracy has not yet biomechanically been investigated in contact combative sports, such as mixed martial arts. Our preliminary result would supply the first “picture” on this factor: weight cutting can significantly decrease the striking accuracy. It should be noted that our test had only static accuracy. In a dynamic situation (e.g., in competitions), the decreased accuracy could be amplified, leading to miss-strikes. It is common knowledge that athletes who land a higher percentage of their strikes can be more efficient and waste less energy on missed techniques. Therefore, weight cutting would not bring advantage for competitions. Again, the preliminary result needs to be justified by future studies.

Like the mixed results in the current literature, the preliminary evidences from this biomechanical investigation could also not be capable of mitigating real risks. The biomechanical results only confirm that weight cutting is a highly complicated issue that is influenced by multi-layer factors and/or numerous confounding variables that researchers have not learnt of. Considering all the current aspects, only one point is clear: weight cutting will continue to co-exist with combat sports. In this scenario, one proposition would be worthy to keep in mind: “what is reasonable is real; that which is real is reasonable” (from the book of Georg Wilhelm Friedrich Hegel: Elements of the Philosophy of Right [42]). The sentence properly describes the paradox which exists in weight cutting practices in current combat sports. Scientifically, we should not (e.g., based on health-risk alone) oppose the weight cutting that has been existed in combat sports, but should investigate any negative effects (controlling/minimizing it) and positive effects (making use of it) instead. Considering the mixed results in the current literature, our preliminary results would imply that two key terms—extreme weight cutting and bio-adaptation—might indicate two relevant directions for future biomechanical studies.

### 4.1. Where Is the Limit of Weight Cutting?

Most of the current negative evidences are linked to extreme weight cutting, ranging from 10% to 17% of body weight [7,8]. The discussion about extreme weight cutting seems to be one that rages on and continues to divide opinion amongst fighters, coaches, fans, and the media. Actually, we are still in the dark as to what the weight cutting limit is and, at least, what the biomechanical effects of weight cutting on performance are.

Weight cuts can vary in severity, with some fighters opting to drop just a few pounds or even become close to their natural weight. Others can partake in brutal weight cuts, losing up to 11% of their body weight [23] in an attempt to have a size advantage over their opponents in competitions. However, our preliminary results indicate that a size advantage does not equal a power/strength advantage.

The large differences of weight cutting that exist in the practice would suggest that there should be a critical value (i.e., a limit) of weight cut. Below the limit, an athlete may prevent the negative effects of weight cutting. However, the large and widespread anthropometrical diversity and athletic/training experience that exists among athletes would restrict a standard approach in defining the limit; instead, an individualized quantification would be significantly practical [43,44]. Therefore, future studies should focus on developing various practical means and methods to supply real-time biomechanical feedback in training to keep weight cutting below the limit in order to prevent negative effects occurring, e.g., a feedback alarm will be given when the reaction time increases over 5% between training sessions. This would be the first step towards safe training. 

### 4.2. How to Positively Alter the Weight-Cut Limit Biomechanically in the Practice?

It is common among combat athletes to engage in some form of weight cutting regularly and, as such, weight cutting is considered to be a necessary part of the sport, and is even viewed as an ability that is required for the sport [16]. Then, a question is raised: is the ability biomechanically entrainable? The answer is yes, through bio-adaptation training. This would be the second step: gradually increase the ability, i.e., the weight cutting limit.

Fundamentally, human physical adaptation is an inherited ability that facilitates survival in hostile environments [45]. In response to some types of repeated external stimuli, the human body is able to modify over time [46] and, as such, bio-adaptation is an ability related to long-time training, i.e., an ability can only be gradually increased [45,46]. To the best of our knowledge, there is no biomechanical study that exists to date to quantify this training process. Future biomechanical studies are needed to quantify the intensity and duration at various competition levels related to bio-adaptation. Consequently, biomechanically-controlled weight cutting training could be integrated into martial arts training practices. Such a biomechanical approach would have the potential to inform practitioners on how to benefit from weight cutting, while minimizing the risks.

Practically, the results of the current study would provide an opportunity with a great potential to initiate real-time biomechanical feedback training that could develop an athletes’ weight cutting ability, i.e., feedback training that aims to identify individual weight cutting limits and positively alter the individual’s weight cutting limit. Biomechanically, the real-time feedback should focus on obtaining the attack power, attack accuracy and reaction time in the training environment and these feedbacks can now be easily obtained by LEDs (stimuli) and IMUs (inertial measurement unit, a wireless small sensor) [47,48,49]. When an IMU is attached to a punching bag, a hand or a foot, the IMU will supply the velocity data of the bag, the hand or the foot during striking. The velocity can be used to estimate the attack power. When two IMUs are synchronously used, the dynamic distance between the two sensors (i.e., accuracy) can be quantified [50]. When multiple IMUs are used, e.g., ≥three on the bag and one on each hand and each foot, together with LEDs, the PNSRT can be determined. Of course, research and development studies are needed for realizing this potential for coaches and practitioners.

This is the first biomechanical study that provides an objective view of weight cutting effects on martial arts performance. It is understandable that there are limitations associated with this study. There are two notable limits which should be carefully considered during the design of future studies. First, due to the real-life data collection and the requirement of the method consistency (i.e., the same data collection technologies), the subjects were limited to one location instead of being international (which could be done if the same technology was available among the collaborators). Although we did our best, the sample size was still very small, and, importantly, the very small sample has a large between-subject deviation of power-related parameters due to confounding variables, such as weight class, amount of weight cut, duration of weight cutting, and the dominant/non-dominant limbs’ strikes. Regardless what type of statistical analysis could be chosen, it would always be possible to argue that the selected statistical test could have the statistical power to validate the outcomes. As such, the current study is essentially a case study with a number of observations. As a case-like study, the results are useful for revealing potentially relevant variables and/or to raise important questions for future researches in the field. It is well known that pilot case studies will promote more replication studies to verify/improve the generalizability of the potentially important results found in these studies. Further, anchored in real-life situations, the current study would have the strength to add a rich and holistic biomechanical account of weight cutting phenomenon. These insights could be construed as tentative hypotheses that could help structure future research and, with great potential, advance the field’s knowledge base. In order to validate these preliminary results, the recruitment of sufficient subjects should be first solved in future studies. Second, the variations in weight class, amount of weight cut, duration of weight cutting, dominant/non-dominant limbs’ strikes, training level and years in a sample could lead to over- or under-estimation of the potential effects. These factors need to be carefully considered in future studies.

Notwithstanding the limitations, the preliminary study has achieved the goals that a pilot study [51] can reach, i.e., to design and test new biomechanical research protocols, to evaluate the feasibility of data analysis, and to supply suggestions, such as sample recruitment strategies and preparations for future studies. Additionally, the pilot study demonstrates that researchers need to do a lot more before acquiring a holistic biomechanical understanding of the effect of weight cutting on martial arts performance. In short, this study could be one of the important stages in the research journey, as it supplies preliminary results for brainstorming, as well as identifies the potential problem areas and deficiencies in the research protocol prior to the implementation of future studies.

## 5. Conclusions

This pilot study applied, for the first time, biomechanical quantification in exploring the effects of weight cutting in real-life competitions. By letting the subjects to use their preferred weight cutting processes, the pilot study has revealed that there is actually weight regain instead of weight loss. The biomechanical quantification has divulged that the weight regain condition can speed up the perceptional reaction, slow down the limbs’ movement, worsen the striking accuracy and, possibly, decrease the strike power. The preliminary results are inconclusive regarding the competitive advantages. While, perhaps, suggesting more questions than answers, the results of this pilot study provide the foundation for further biomechanical explorations of the effects of weight cutting on martial arts performance. 

## Figures and Tables

**Table 1:** Participants’ Information.

Participants’ Information	Rang	µ ± SD
Body weight (kg)	65.6–96.1	78.8 ± 9.9
Body height (m)	1.71–1.88	1.79 ± 0.06
Age (years)	20–32	25.7 ± 3.9
Years of training	1.5–9.0	5.1 ± 3.0
Years of competition	0–6	2.9 ± 2.2

µ: mean, SD: standard deviation.

**Table 2:** Results of weight cutting of all subjects.

Weight-Cut Process	Subj.1	Subj.2	Subj.3	Subj.4	Subj.5	Subj.6	Subj.7	µ ± SD
Weight before cut process (kg)	65.6	70.8	74.8	78.5	81.3	84.3	96.1	78.8 ± 9.9
Weight loss (kg)	1.6	4.3	3.9	2.4	4.2	3.6	4.8	3.5 ± 1.1
% change of weight loss	2.4%	6.1%	5.2%	3.1%	5.2%	4.3%	5.0%	4.5% ± 1.3%
Weight regain after weigh-in (kg)	2.4	4.4	5.2	2.9	5.1	5.4	4.1	4.2 ± 1.2
% change of weight regain	3.7%	6.2%	7.0%	3.7%	6.3%	6.4%	4.3%	5.4% ± 1.4%
Weight after cut process (kg)	66.4	70.9	76.1	79.0	82.2	86.1	95.4	79.4 ± 9.7
% change after cut process	1.2%	0.1%	1.7%	0.6%	1.1%	2.1%	−0.7%	0.9% ± 1.0%

Subj.: subject, µ—mean, SD: standard deviation.

**Table 3:** The influences of weight cutting on individual performance.

Biomechanical Parameters		Subj.1	Subj.2	Subj.3	Subj.4	Subj.5	Subj.6	Subj.7
Reaction (s)	CNSRT	P	P	P	P	P	P	P
	PNSRT	N	N	N	N	None	N	N
	Total	P	P	P	P	P	P	P
Max Power (Watt)	Linear	P	N	P	N	N	P	N
	Rotary	P	N	N	N	P	P	N
	Total	P	N	N	N	P	P	N
Average Power (Watt)	Linear	P	N	P	P	N	N	N
	Rotary	P	N	N	N	P	P	N
	Total	P	N	N	N	P	P	N
Accuracy (mm)		N	N	P	N	N	P	N

CNSRT: central nervous system response time, PNSRT: peripheral nervous system response time, Subj.—subject, P: positive effects, N: negative effect.

**Table 4:** Results of the overall effects of weight cutting.

Biomechanical Parameters		Pre	Post	% Change
Reaction (s)	CNSRT	0.54 ± 0.21	0.44 ± 0.15	−18.52% **
	PNSRT	0.26 ± 0.15	0.31 ± 0.12	19.23% **
	Total	0.80 ± 0.20	0.75 ± 0.18	−6.25% **
Max Power (Watt)	Linear	664.52 ± 524.15	1745.19 ± 2920.44	
	Rotary	2720.52 ± 2052.29	3033.56 ± 2613.11	
	Total	3385.57 ± 3173.17	4776.82 ± 5183.93	
Average Power (Watt)	Linear	424.99 ± 323.28	836.45 ± 1257.93	
	Rotary	1789.45 ± 2211.60	1736.27 ± 2039.30	
	Total	2214.44 ± 2272.02	2572.72 ± 3409.67	
Accuracy (mm)		97.68 ± 67.24	104.97 ± 74.31	7.46% *

CNSRT: central nervous system response time, PNSRT: peripheral nervous system response time. **: *p* < 0.01, *: *p* < 0.05.

## Data Availability

The data presented in this study are available on request and after appropriate IRB approvals.
